# Identification of an Invertase With High Specific Activity for Raffinose Hydrolysis and Its Application in Soymilk Treatment

**DOI:** 10.3389/fmicb.2021.646801

**Published:** 2021-04-08

**Authors:** Juanjuan Liu, Jing Cheng, Min Huang, Chen Shen, Ke Xu, Yazhong Xiao, Wenjuan Pan, Zemin Fang

**Affiliations:** ^1^School of Life Sciences, Anhui University, Hefei, China; ^2^Anhui Key Laboratory of Modern Biomanufacturing, Hefei, China; ^3^Anhui Provincial Engineering Technology Research Center of Microorganisms and Biocatalysis, Hefei, China; ^4^Anhui RenRenFu Bean Co., Ltd., Hefei, China

**Keywords:** invertase, soymilk pretreatment, raffinose hydrolyzation, melibiose, gut microbiota

## Abstract

The hydrolyzation of raffinose into melibiose by using invertases under mild conditions improves the nutritional value of soybean products. However, this strategy has received little attention because a suitable invertase remains lacking. In this study, a novel invertase named InvDz13 was screened and purified from *Microbacterium trichothecenolyticum* and characterized. InvDz13 was one of the invertases with the highest specific activity toward raffinose. Specifically, it had a specific activity of 229 U/mg toward raffinose at pH 6.5 and 35°C. InvDz13 retained more than 80% of its maximum activity at pH 5.5–7.5 and 25–40°C and was resistant to or stimulated by most cations that presented in soymilk. In soymilk treated with InvDz13 under mild conditions, melibiose concentration increased from 3.1 ± 0.2 to 6.1 ± 0.1 mM due to raffinose hydrolyzation by InvDz13. Furthermore, the prebiotic property of InvDz13-treated soymilk was investigated via *in vitro* fermentation by human gut microbiota. Results showed that InvDz13 treatment increased the proportion of the beneficial bacteria *Bifidobacterium* and *Lactobacillus* by 1.6- and 3.7-fold, respectively. By contrast, the populations of *Escherichia* and *Collinsella* decreased by 1.8- and 11.7-fold, respectively. Thus, our results proved that the enzymatic hydrolysis of raffinose in soymilk with InvDz13 was practicable and might be an alternative approach to improving the nutritional value of soymilk.

## Introduction

Raffinose (α-D-galactopyranosyl-[1→6]-α-D-glucopyranosyl-[1→2]-β-D-fructofuranoside) acc- ounts for the second-highest concentration of galacto-oligosaccharides in soybean. Raffinose concentration in legume ranges from 4.8 to 20.1 mg/g dry matter on the basis of seed sources (Hartwig et al., 1997). It has been recognized as a candidate human prebiotic because it promotes the growth of beneficial microbes, such as *Bifidobacterium* and *Lactobacillus*, in the gut (Trojanová et al., 2006; Gibson et al., 2017; Adamberg et al., 2018; Zartl et al., 2018). However, raffinose leads to flatulence in humans due to its utilization by gut bacteria, such as *Escherichia*, *Enterococcus*, and *Streptococcus* (Mao et al., 2018). Many studies have evaluated the effectiveness of different processing techniques, including soaking, cooking, germination, and enzymatic hydrolyzation, for raffinose removal from soybeans (Oboh et al., 2000; Medeiros et al., 2018). Among these techniques, enzymatic hydrolysis using α-galactosidases to hydrolyze raffinose into galactose and sucrose is thought to be a promising strategy because raffinose is an α-galactosyl derivative of sucrose (Huang et al., 2018; Jang et al., 2019; Katrolia et al., 2019; Geng et al., 2020). Furthermore, enzymatic reactions are frequently conducted under mild energy- and cost-saving conditions (Jang et al., 2019).

Raffinose is a conjunction of melibiose and fructose. Melibiose is a reducing disaccharide that is composed of galactose and glucose with α-1,6 linkages (Xu et al., 2017). Melibiose has gained considerable attention since the early 21st century because of its beneficial attributes. For example, it promotes calcium absorption in the intestines and helps cure atopic dermatitis (Kaneko et al., 2004). Evidence suggests that melibiose is a novel autophagy-inducing small molecule that inhibits aggregation-mediated neurodegenerative disorders, including Alzheimer’s and Parkinson’s diseases, as well as polyglutamine-mediated diseases (Lee et al., 2015; Rusmini et al., 2019; Lin et al., 2020). Melibiose is a disaccharide that is indigestible by humans. It can be used as a high-value additive in human functional foods and pharmaceuticals to maintain and promote good health (Tanaka et al., 2016). The appropriate intake of melibiose increases *Bifidobacterium* growth and improves stool condition in healthy humans (O’Connell et al., 2013; Adamberg et al., 2018). In contrast to the hydrolysis of raffinose into sucrose and galactose, the hydrolysis of raffinose into melibiose improves the nutritional value of soybean and soymilk, which contains 8.4–30 mg/g dry matter of raffinose (Garro and Savoy, 2012). Several studies have focused on the enzymatical hydrolysis of raffinose into melibiose. For example, an engineered *Saccharomyces cerevisiae* was constructed to improve the whole-cell biocatalytic production of melibiose from raffinose (Zhou et al., 2017). However, no investigation was conducted to hydrolysis of raffinose into melibiose in soybean and soymilk, and no work has paid attention to evaluating the effect of the end-products of this process on human health, especially gut microbial diversity.

Invertases (EC 3.2.1.26) are carbohydrases that catalyze the hydrolysis of sucrose, raffinose, and other related glycosides (Kotwal and Shankar, 2009). The direct hydrolysis of raffinose into melibiose and fructose by using invertase is a simple way to obtain melibiose in soymilk. Commercially, soymilk is obtained by soaking and grinding soybeans with tap water. The crude slurry is then filtered, boiled, and kept at boiling temperature for about 5–15 min. Finally, the heated soymilk was quickly cooled to room temperature to obtain soymilk (Zuo et al., 2016). The invertases added into soymilk are recommended to complete raffinose hydrolysis at moderate or lower temperatures to facilitate soymilk preparation. Furthermore, given that soymilk has a pH of approximately 5.5–7.0 and is rich in cations (Jiang et al., 2013; Zuo et al., 2016), the invertases used in soymilk preparation must show high activity toward raffinose under mild conditions. However, the raffinose hydrolysis capability of only several invertases have been evaluated (Cuezzo de Ginés et al., 2000; Zhou et al., 2016; Xu et al., 2017). Characterized invertases, such as invertases from *Penicillium chrysogenum* sp. 23 (Yakimova et al., 2017) and pea seedlings (Kim et al., 2011), have limited utility in raffinose hydrolyzation because of their low activities (highest activity of approximately 10% under optimal conditions) and specific activities toward raffinose (<50 U/mg) under mild conditions (pH 5.5–7.0 and ambient or low temperatures). Hence, discovering novel invertases with high raffinose hydrolysis activity under mild conditions will help hydrolyze raffinose into melibiose in soybean products.

Microbial sources that thrive in cold environments, such as the Antarctic, have attracted considerable attention because their hydrolytic enzymes typically have higher activity at lower temperatures than the hydrolytic enzymes of microbes from temperate environments (Perfumo et al., 2018). In the present study, a novel invertase from GH68 was screened and characterized. Its application in hydrolyzation and improving the prebiotic property of soymilk was also evaluated. Our results demonstrated that InvDz13 was one of the invertases with the highest specific activity toward raffinose and was resistant to or even stimulated by most cations in soymilk. InvDz13-treated soymilk increased the proportion of the beneficial bacteria and decreased the populations of *Escherichia* and *Collinsella*. Therefore, InvDz13 is suitable for hydrolyzing raffinose into melibiose in soymilk under mild conditions to improve the prebiotic property of soymilk.

## Materials and Methods

### Screening for Positive Clones With Invertase Activity

Antarctic sediment soil (S 62°8′7.8″, W 58°58′50.03″) was collected in Dec. 2016 and stored at −20°C until use. One gram of wet sediment was mixed with 9 mL of sterilized seawater and shaken at 200 rpm and 15°C for 2 h. The suspension was diluted through the standard dilution-to-extinction method to 10^–6^. Then, 100 μL aliquots of the dilutions were spread on agar screening plates containing 0.2% raffinose, 0.5% tryptone, 0.1% yeast extract, 3.3% synthetic sea salt, and 1.0% agar and incubated at 16°C for 7 days. Colonies grown on screening plates were picked and cultured in liquid screening medium in 24-well plates at 16°C for 3 days. Then, supernatants were withdrawn and used for invertase activity determination with raffinose as the substrate. Samples with high activities were used for further research.

### Identification of the Invertase-Producing Strain

Positive strains were cultured in 5 mL of standard synthetic sea salt medium (Sigma-Aldrich, St. Louis, MO, United States) and incubated at 16°C on a rotary shaker at 180 rpm for 24 h. Then, the cells were withdrawn, and total genomic DNA was extracted in accordance with the manufacturer’s instructions (Sangon Biotech, Shanghai, China) and used as the template. The 16S rRNA gene was amplified by using the eubacteria primers of Bact-27F (5′-AGAGTTTGATCMTGGCTCAG-3′) and Bact-1492R (5′-GGTTACCTTGTTACGACTT-3′). The PCR products were cloned into the pGEM-T vector (Promega, WI, United States) and sequenced (Sangon Biotech, Shanghai, China). A Blast search of NCBI^1^ was performed to determine the most closely related species.

### Purification of InvDz13 From the Culture Supernatant

After culturing the strain Dz13 in 1 L Erlenmeyer flasks containing 400 mL of liquid screening medium at 200 rpm and 16°C for 72 h, the culture supernatant was withdrawn by centrifugation at 10,000 × *g* for 5 min and partially purified with a DEAE-Sepharose FF column (10 mm × 200 mm, Amersham Pharmacia, Uppsala, Sweden). The column was pre-equilibrated with citrate–phosphate buffer and eluted with a linear gradient of NaCl (0–1 M in a citrate–phosphate buffer with the flow rate of 0.8 mL/min). The fractions exhibiting invertase activity were pooled, concentrated in a low-binding regenerated cellulose membrane, and further purified via gel filtration through Sephacryl S100 (Amersham Pharmacia) pre-equilibrated with 50 mM citrate–phosphate buffer (pH 6.5), 1 mM EDTA, 10% (v/v) glycerol, 5 mM β-mercaptoethanol, and 150 mM NaCl. The purified invertase was designated as InvDz13.

### Identification and Sequence Analysis of InvDz13

The purified InvDz13 was identified by using LC–ESI–MS/MS (LTQ, Thermo Fisher Scientific, Shanghai, China) and mapped to the GenBank database. On the basis of strain and protein identification results, the InvDz13 gene was then cloned from the genome of strain Dz13 by using the InvF (5′-ATGCACAC TCCCCCGAAG-3′) and InvR (5′-TCAGGGCAGCGGCGTG ACC-3′) primers designed with the levansucrase gene from *Microbacterium trichothecenolyticum* as the reference. The PCR product was sequenced by Sangon Biotech (Shanghai, China).

The sequence similarity search of InvDz13 was performed by using BlastP at NCBI^2^. The enzyme’s module structure was analyzed with the simple modular architecture research tool SMART^3^. The multiple sequence alignment of InvDz13 with other related invertase sequences was performed by using Clustal X 2.0 and GeneDoc^4^.

### Invertase Activity Assay

Protein samples were diluted in a suitable volume of citrate–phosphate buffer (50 mM, pH 6.5). Invertase activities were measured in 1 mL reaction mixtures containing 20 μL of the purified enzyme, 50 mM citrate–phosphate buffer (pH 6.5), and 200 mM sucrose and incubated at 35°C for 5 min. The reaction was terminated by heating the assay mixture at 100°C for 5 min. The amounts of released glucose and fructose were measured by using the 3,5-dinitrosalicylic acid method (Ashwell and Hickman, 1957). The unit (U) of invertase activity was defined as the amount of enzyme required to hydrolyze 1 μmol of sucrose per min under assay conditions.

### Biochemical Characterization

The homogeneity of the target protein was determined through sodium dodecyl sulfate polyacrylamide gel electrophoresis (SDS-PAGE) in 12% polyacrylamide gel and stained with Coomassie brilliant blue R250. Protein concentration was assayed by using the Bradford method at 595 nm with bovine serum albumin as the standard (Sangon Biotech). The SDS-PAGE gel was washed with 50 mM citrate–phosphate buffer at pH 6.5 for 1 h to remove SDS for native-PAGE analysis. It was then incubated in an acetate–phosphate buffer (50 mM, pH 6.5) containing 200 mM sucrose at 35°C for 30 min and actively stained with 100 mM NaOH solution containing 0.2% triphenyl tetrazolium chloride after sucrose solution removal (Van et al., 2013).

The effect of pH on enzymatic activity was determined at 35°C in 50 mM citrate–phosphate buffer (pH 4.5–8.5) and 50 mM Tris–HCl buffer (pH 8.5–9.5). The effect of temperature on enzymatic activity was determined at pH 6.5 and temperatures ranging from 10–55°C. Enzyme stabilities against pH and temperature were determined by incubating proteins at various temperatures and different pH values. Residual activities were determined as mentioned above. All experiments were performed in triplicate.

The effects of metal ions, including Na^+^, K^+^, Mg^2+^, Cd^2+^, Sr^2+^, Cu^2+^, Ca^2+^, Mn^2+^, Co^2+^, Zn^2+^, Ni^2+^, Fe^2+^, and Cr^2+^, on InvDz13 activity were investigated in the presence of 5 mM each ion at pH 6.5 and 35°C by using sucrose and raffinose as the substrates.

### Kinetic Analysis

The appropriate concentration of InvDz13 was utilized under optimal conditions to determine kinetic parameters (*K*_m_, *V*_max_, and *k*_cat_/*K*_m_). The reaction was carried out by incubating the enzyme in 50 mM citrate–phosphate buffer (pH 6.5) containing sucrose, raffinose, or stachyose at concentrations of 1–1,000 mM at 35°C for 5 min. The amount of released glucose was quantified by using the glucose oxidase method (Rongsheng Biotech, Shanghai, China). The kinetic constants and their corresponding errors were calculated by fitting the measured rate to the Michaelis–Menten equation with the computer program Origin 8.0 (*n* = 9).

### Carbohydrate Assay

The reaction supernatants were collected after 10 min of reaction and used to determine the mono-, di-, and tri-saccharides released from sucrose, raffinose, or stachyose after InvDz13 addition. Saccharides were determined by using high-performance liquid chromatography (HPLC). Briefly, 50 μL of each sample was analyzed at 30°C by using a TSKgel Amide-80 column (4.6 mm × 250 mm, 5 μm, Tosoh Corporation, Kyoto, Japan) and an evaporative light-scattering detector 2424 (Waters, United States). The eluting buffer was acetonitrile:water (70: 30, v/v) at the flow rate of 0.4 mL/min.

### Treatment of Soybean Milk With InvDz13

Soybeans harvested in northeast China were obtained from Anhui RenRenFu Bean Co., LTD and soaked in water (water: dry soybean, 3:1, w/w) for 8 h at 20°C. The soaked soybeans were ground with water at a total ratio of 1:7 by using a colloid mill for three passes. Subsequently, the crude slurry was filtered through muslin cloth to obtain the crude soymilk. Boiled soymilk was prepared by boiling the crude soymilk for 15 min. The enzymatic hydrolysis of saccharides in soymilk was performed as follows: InvDz13 at a final concentration of 10 U/mL was added into 100 mL of crude or boiled soymilk. The reaction was conducted at 30°C for 1 h on a shaker at 100 rpm. The crude soymilk was further boiled for 15 min after treatment with InvDz13. Five milliliters of each soymilk sample were mixed with 70% ethanol (1:1, v/v) for 5 min. Then, the samples were centrifuged at 20,000 × *g* to discard proteins. The supernatant was recovered and filtered through 0.22 μm filters. The saccharides were determined through HPLC as described above.

### *In vitro* Fermentation of Human Fecal Samples With Soymilk and InvDz13-Treated Soymilk

Fresh fecal samples were obtained from three physically and mentally healthy adult donors (two females and one male) who volunteered to participate in the experiment. All donors were 20 years old, were on a regular diet, and did not have gastrointestinal diseases or undergone antibiotic treatment within 3 months. The same amount of fecal sample from each donor was promptly suspended in pre-prepared sterile physiological saline (0.9%, w/v) and blended to yield 15% (w/v) fecal slurry. After 5 min of centrifugation at 500 × *g* for 5 min, the suspension was diluted 10 times with gut microbiota medium [1.0 L, containing 2 g of tryptone peptone, 2 g of yeast extract, 0.02 g of hemin, 0.5 g of L-cysteine, 0.5 g of bile salts, 0.1 g of NaCl, 0.04 g of K_2_HPO_4_, 0.04 g of KH_2_PO_4_, 0.01 g of MgSO_4_⋅7H_2_O, 0.01 g of CaCl_2_⋅6H_2_O, 2 g NaHCO_3_, 1.0 mL of resazurin solution (1%, w/v), 2.0 mL of Tween-80, and 10 μL of vitamin K] (Di et al., 2018; Control group) or gut microbiota medium containing 1/5 (v/v) soymilk (RAF group) or InvDz13-treated soymilk (M + F group). All samples were incubated under anaerobic conditions at 37°C for 24 h, removed from incubation, submerged in an ice bath to halt microbial activity, and subjected to DNA analysis. Each experiment was replicated independently three times.

### DNA Extraction and 16S rRNA Gene Amplicon Analysis

Genomic DNA from different fermentative fecal samples was extracted by using an E.Z.N.A. ^®^Stool DNA Kit (D4015, Omega, Inc., United States) in accordance with the manufacturer’s instructions. The V3–V4 region of the prokaryotic 16S rRNA gene was amplified with slightly modified versions of primers 341F (5′-CCTACGGGNGGCWGCAG-3′) and 805R (5′-GACTACHVGGGTATCTAATCC-3′) in a two-step procedure to limit amplification bias. The final amplified products were purified by using AMPure XT beads (Beckman Coulter Genomics, Danvers, MA, United States) and quantified with Qubit (Invitrogen, United States). The amplicon pools were prepared for sequencing. The amplicon library’s size and quantity were assessed by using an Agilent 2100 Bioanalyzer (Agilent, United States) and a Library Quantification Kit for Illumina (Kapa Biosciences, Woburn, MA, United States), respectively. The samples were sequenced on an Illumina MiSeq platform in accordance with the manufacturer’s recommendations provided by LC-Bio (Hangzhou, China).

Raw sequence reads were quality-filtered in accordance with Fqtrim (v0.94). Chimeric sequences were filtered by using Vsearch software (v2.3.4). Sequences with ≥97% similarity were assigned to the same operational taxonomic units (OTUs) by Vsearch (v2.3.4). Ribosomal Database Program (classifier) was used for the taxonomic classification of sequences and assignment to particular clusters. The differences in the dominant species in different groups and multiple sequence alignments were determined by using Mafft software (v7.310) to study the phylogenetic relationship of different OTUs. OTU abundance information was normalized by using the standard sequence number corresponding to the sample with the lowest number of sequences. The alpha diversity of samples was analyzed and calculated by using QIIME (v1.8.0). Beta diversity was calculated through PCoA and cluster analysis with QIIME software (v1.8.0). Taxonomic changes that differed significantly between different groups were analyzed through linear discriminant analysis effect size (LEfSe) algorithm by using the software LEfSe 1.0.

### Statistical Analysis

All of the experimental data were presented as mean ± standard deviation. Statistical significance was evaluated through one-way ANOVA followed by Student’s *t*-test with GraphPad Prism 7.0. *P* < 0.05 was considered statistically significant.

## Results and Discussion

### Screening and Sequence Analysis of InvDz13

An invertase from strain Dz13 (defined as InvDz13) with high specific activity toward raffinose (ca., 130 U/mg) was screened out through the primary screening of invertase-producing bacteria on agar plates, the rescreening of high-activity invertase by using liquid fermentation, and the testing of invertase activity in the culture supernatant. The 16S rRNA gene of strain Dz13 shared 99.93% sequence identity (100% coverage) and 99.93% sequence identity (99% coverage) with that of an uncultured bacterium clone CZ121 (Accession No. GU272293) and *Microbacterium* sp. strain L4 (KY412841) in the GenBank database, respectively. It shared 99.1% sequence identity (100% coverage) with an *M. trichothecenolyticum* type strain in the EZbiocloud database (Accession No. JYJA01000006). Given that 98.65% 16S rRNA gene sequence similarity could be used as the threshold for differentiating two bacterial species (Kim et al., 2014), Dz13 was thus named temporarily as *M. trichothecenolyticum* Dz13.

**FIGURE 1:**
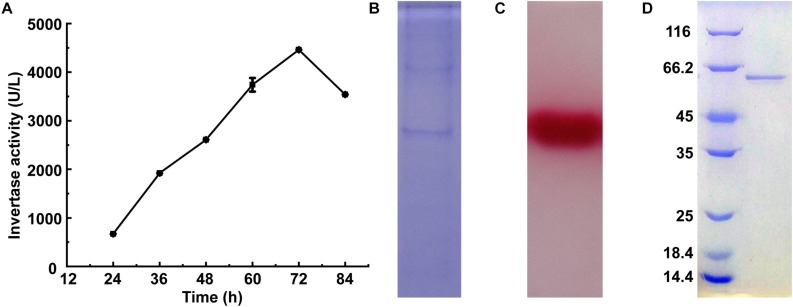
Expression and purification of InvDz13. **(A)** Time course of invertase activity in culture supernatant of *M. trichothecenolyticum* Dz13. **(B,C)** Coomassie brilliant blue **(B)** and triphenyl tetrazolium chloride **(C)** stained 15% SDS-PAGE gel of InvDz13 in culture supernatant of *M. trichothecenolyticum* Dz13. **(D)** Coomassie brilliant blue stained 15% SDS-PAGE gel of InvDz13 after ion-exchange column and gel filtration column purification.

Invertase activity reached 4,460 U/L after 72 h of the cultivation of *M. trichothecenolyticum* Dz13 in liquid screening medium (Figure 1A). Several bands were observed in the SDS-PAGE gel loaded with the culture supernatant withdrawn at 72 h (Figure 1B). However, triphenyl tetrazolium chloride staining results showed only one band with invertase activity (Figure 1C; Van et al., 2013). This protein, which was named InvDz13, was further purified successfully from the culture supernatant by using an ion-exchange column and gel filtration column (Figure 1D) and was identified through the LC–TOF–MS/MS technique. InvDz13 was matched to a levansucrase from *M. trichothecenolyticum* with 99% probability (KJL40835). Only three strains from genus *Microbacterium*, including *Microbacterium paraoxydans* (Ojha et al., 2016), *Microbacterium laevaniformans* (Kim et al., 2005), and *Microbacterium saccharophilum* K-1 (Ohta et al., 2014), have been reported to produce beta-fructofuranosidase or levansucrase. Therefore, our research on invertase from *M. trichothecenolyticum* Dz13 will deepen our understanding of invertases from *Microbacterium* spp.

The InvDz13 gene was cloned by using the *M. trichothecenolyticum* Dz13 genome as the template and two primers designed with the levansucrase gene from *M. trichothecenolyticum* as the reference. The cloned InvDz13 was 537 aa in length. It shared the same sequence with a GH68 protein from *M. trichothecenolyticum* (WP_045301577) and 89.49–96.46% sequence identities with other GH68 proteins from the GenBank database. All these proteins are annotated from many different RefSeq genomes and have not been biochemically characterized^5^. InvDz13 possessed the Pfam signature of Glyco-hydro-68 from residues 60 to 522 (*E*-value 3.1 × e-145). In InvDz13, a signal peptide was predicted from residues 1 to 35 in accordance with the detection of invertase activity in the culture broth. Comparison with the amino acid sequences of the characterized invertases revealed that InvDz13 had 11 specific conserved regions of the GH68 family^6^. Therefore, InvDz13 was a member of the GH68 enzyme family.

### Biochemical Characterization of InvDz13

**FIGURE 2:**
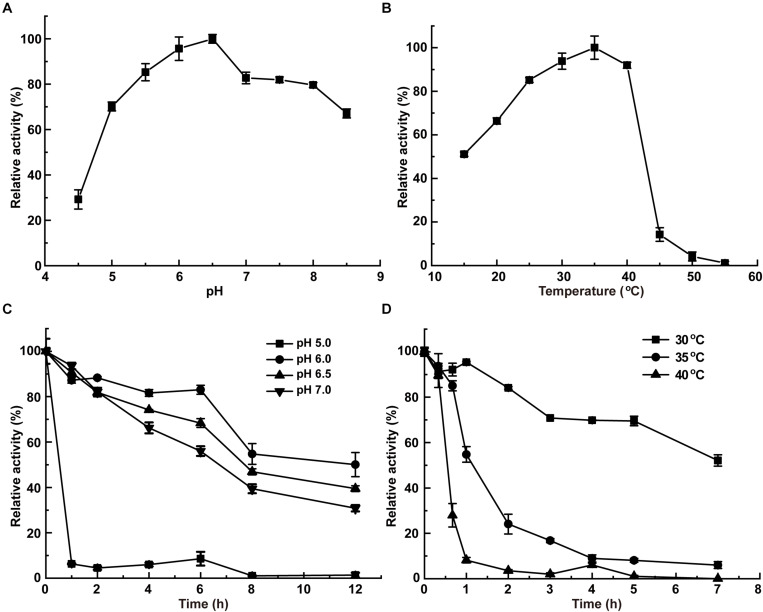
Effects of pH and temperature on the activity and stability of InvDz13. **(A)** pH optimum. Samples were incubated at 35°C. **(B)** Temperature optimum. Samples were incubated at pH 5.0. **(C)** pH stabilities at pH 5.0, 6.0, 6.5, and 7.0, respectively. Samples were incubated at 30°C. **(D)** Thermostabilities at 30, 35, and 40°C, respectively. Samples were incubated at pH 6.5. Standard deviations and values were calculated from triplicate technical repeats of measurements.

InvDz13 displayed maximum activity at pH 6.5 when sucrose was used as the substrate and retained more than 70% of its maximum activity at pH 5.0–8.5 (Figure 2A). The optimal temperature of InvDz13 was 35°C. InvDz13 retained 50% of its original activity when tested at 15°C and 10% of its highest activity when tested at 45°C (Figure 2B). The optimal pH and temperature of InvDz13 were similar to those of bacterial invertases from *Bacillus* sp. HJ14 (Zhou et al., 2016; Table 1), *Arthrobacter globiformis* (Win et al., 2004), and *Bifidobacterium infantis* (Warchol et al., 2002), which have optimal pH values and temperatures of 6.0–7.5 and 30–40°C, respectively.

**TABLE 1:** Comparison of biochemical properties of InvDz13 with other bacterial invertases.

Microorganism	Opt. pH	Opt. temp. (°C)	*K*_m_ (mM)		Specific activity (U/mg)		References
>						
			Sucrose	Raffinose	Sucrose	Raffinose	
*Microbacterium trichothecenolyticum*	6.5	35	4.5 ± 0.2	14.2 ± 0.7	225	229	This study
*Synechocystis* sp.	7.0	30	14.7	NR^#^	NR	NR	Kirsch et al., 2018
*Leuconostoc mesenteroides*	6.0	45	25.66 ± 1.2	56.82 ± 1.5	469 ± 23	195 ± 16	Xu et al., 2017
*Bacillus* sp. HJ14	8.0	30–32.5	62.9	NR	155.1 ± 1.5	4.5 ± 0.1	Zhou et al., 2016
*Microbacterium saccharophilum* K-1	6.5	40	NR	NR	140.1 ± 14.1	NR	Ohta et al., 2014
*Bifidobacterium longum* KN29.1	6.2	50	29.4 ± 1.5	NR	106.09	NR	Jedrzejczak-Krzepkowska and Stanislaw Bielecki, 2011
*Bifidobacterium adolescentis* G1	5.7	50	38	79.4	86	NR	Omori et al., 2010
*Enterobacter* pCNK4	8.0	37	NR	NR	0.438	NR	Kumar et al., 2016
*Enterobacter* pCNK5	8.0	37	NR	NR	0.184	NR	Kumar et al., 2016
*Thermotoga maritima*	5.5	60	51	NR	NR	NR	Menéndez et al., 2013
*Microbulbifer* rFF33	6.0	35	NR	NR	685.6	150.83	Kobayashi et al., 2012
*Microbulbifer* rIN33	6.0	35	NR	NR	30	6.6	Kobayashi et al., 2012
*Bifidobacterium longum*	6.2	37	31.45	64.56	NR	NR	Bujacz et al., 2011
*Leishmania*	5–7	40	152 ± 30	141 ± 30	NR	NR	Belaz et al., 2015
*Bifidobacterium adolescentis* G1	6.1	45	11	NR	101	NR	Muramatsu et al., 2014

*^#^NR: not reported.*

The pH stability of InvDz13 was assayed at 30°C. InvDz13 was stable at pH 6.0 and retained approximately 50% of its original activity after 8 h of incubation at the optimal pH of 6.5 (Figure 2C). It lost its activity quickly after 1 h of incubation at pH 5.0. InvDz13 was stable at temperatures lower than 30°C with a half-life time of more than 7 h. By contrast, its half-life times at 35°C and 40°C were 1.2 and 0.5 h, respectively, (Figure 2D). It became inactive after incubation at 45°C for 30 min. These data indicated that InvDz13 was a psychrophilic invertase. pH and thermal stabilities are important commercially profitable features of an enzyme given that the operation of enzyme-catalyzed reactions at moderate temperatures and weak acidic/neutral pH reduces energy and equipment costs (Singh et al., 2018).

The effects of cations on InvDz13 activity were evaluated by using sucrose and raffinose as the substrates (Table 2). Overall, cations showed similar effects on InvDz13 activity regardless of the substrate used. Most of the commonly used cations, such as Na^+^, K^+^, Mg^2+^, Ca^2+^, Zn^2+^, and Cr^3+^, had little effect on enzyme activity, with 10% stimulatory or inhibitory effects at the concentration of 5 mM. The cations Fe^2+^, Fe^3+^, and Mn^2+^ increased invertase activity to 169.5, 148.8, and 432.6%, respectively. The capability of the metal ion Mn^2+^ to increase the enzymatic activity of several invertases has been reported. For example, Mn^2+^ enhances the activity of invertase from *Aspergillus phoenicis* by up to 277% (Rustiguel et al., 2015). Mn^2+^ increases INVA and INVB activities by 80% and 20%, respectively, (Pérez de los Santos et al., 2016). The tolerance of InvDz13 to these commonly used ions suggested that it could hydrolyze substances containing various ions, such as soymilk. Cu^2+^ was the only ion that inhibited InvDz13 activity severely. Specifically, only 30–40% activity was retained in the presence of 5 mM Cu^2+^, suggesting that thiol groups or His residues that are important for enzyme activity were present. Cu^2+^ may coordinate with His residues on protein groups and induce conformational changes in protein structure (Pérez de los Santos et al., 2016). Furthermore, Cu^2+^ oxidizes cysteine residues in proteins and cause structural changes and protein activity alterations.

**TABLE 2:** Effects of cations on InvDz13 activity.

Metal ions	Sucrose	Raffinose
None	100.0	100.0
K^+^	94.5 ± 0.9	104.0 ± 0.9
Na^+^	97.5 ± 2.7	106.8 ± 5.0
Sr^2+^	108.8 ± 0.2	111.3 ± 0.8
Cu^2+^	41.9 ± 0.2	33.5 ± 1.9
Fe^3+^	148.8 ± 3.2	128.8 ± 1.5
Mg^2+^	97.4 ± 0.7	82.1 ± 1.3
Ca^2+^	94.9 ± 7.9	89.0 ± 1.2
Co^2+^	169.5 ± 0.8	169.3 ± 3.3
Mn^2+^	432.6 ± 16.5	152.6 ± 1.3
Zn^+^	100.5 ± 4.6	101.9 ± 0.4
Ni^2+^	95.5 ± 4.3	85.7 ± 2.9
Fe^2+^	128.2 ± 0.3	123.0 ± 0.6
Cr^3+^	103.4 ± 1.0	91.4 ± 1.3

*Samples were incubated in the presence of 5 mM each ion at pH 6.5 and 35°C using sucrose and raffinose as the substrates, respectively.*

### Substrate Specificity and Kinetic Constants

**FIGURE 3:**
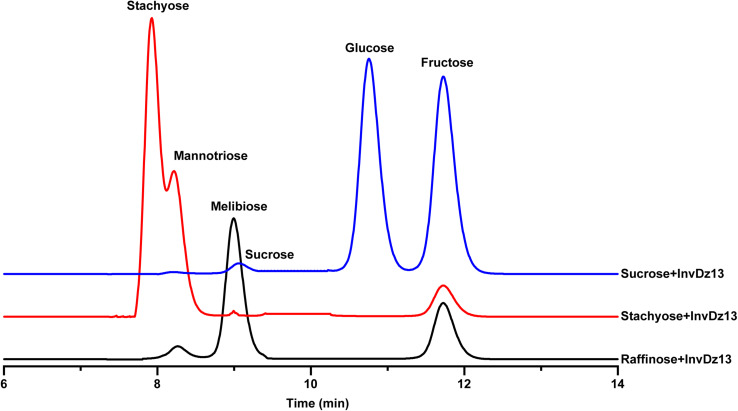
Bioconversion of sucrose, raffinose, or stachyose through InvDz13 hydrolysis. Reaction supernatants were collected after 10 min reaction and used to determine mono-, di-, and tri-saccharides released from sucrose, raffinose, or stachyose after adding InvDz13. Saccharides were determined using HPLC at 30°C by using a TSKgel Amide-80 column and an evaporative light-scattering detector 2424.

The substrate specificity and action mode of InvDz13 were investigated by incubating the enzyme with sucrose, cellobiose, maltose, lactose, raffinose, and stachyose at pH 6.5 and 35°C. In contrast to most bacterial invertases that were highly specific for sucrose (Table 1), purified InvDz13 had specific activities of 225, 229, and 24 U/mg for sucrose, raffinose, and stachyose, respectively. InvDz13 released fructose from sucrose, raffinose, and stachyose (Figure 3) but failed to hydrolyze other saccharides, including cellobiose, maltose, and lactose, because they lacked the β-D-fructofuranosyl moiety (Supplementary Table 1), suggesting that InvDz13 was an invertase (Zhang et al., 2015). Only a few bacterial invertases show activities toward other saccharides, such as raffinose (Table 1). Similar to InvDz13, *Lactobacillus reuteri* CRL 1100 invertase was active on sucrose, raffinose, and stachyose. However, it only showed sucrose activities of 29% and 23% toward raffinose and stachyose, respectively, (Cuezzo de Ginés et al., 2000). Some other reports have shown approximately 10% sucrose activity toward raffinose (Table 1). The activities of bacterial invertases toward stachyose have been rarely reported (Lincoln and More, 2018).

The kinetic constants of rInvDz13 on sucrose were tested under optimal conditions. The values of the kinetic parameters *K*_m_, *k*_cat_, and *k*_cat_/*K*_m_ were 4.5 ± 0.2 mM, 504 ± 0.1 s^–1^, and 112 ± 1.3 mM^–1^ s^–1^, respectively, (Supplementary Figure 1A). The *K*_m_ value of InvDz13 fell at the lower end of the *K*_m_ values of 2.4–370 mM reported for most bacterial invertases, suggesting that the sucrose affinity of InvDz13 was stronger than that of most bacterial invertases (Table 1). For example, invertase from *Synechocystis* sp. shows a *K*_m_ of 14.7 mM toward sucrose (Kirsch et al., 2018), whereas invertases from *Erwinia amylovora* (Bogs and Geider, 2000) and *Bacillus cereus* TA-11 (Yoon et al., 2007) have *K*_m_ values of 125 and 370 mM, respectively.

The kinetic parameters *K*_m_, *k*_cat_, and *k*_cat_/*K*_m_ of InvDz13 toward raffinose and stachyose were 14.2 ± 0.7 mM, 3944 ± 15.3 s^–1^, 277 ± 27.6 mM^–1^ s^–1^, 65.2 ± 3.07 mM, 2368.3 ± 7.1 s^–1^, and 36.3 ± 5.6 s^–1^, respectively, (Supplementary Figures 1B,C), suggesting that InvDz13 had better affinity for sucrose than for raffinose and stachyose. However, the raffinose and stachyose affinity of InvDz13 could not be compared with that of other bacterial invertases because the *K*_m_ values of only two bacterial invertases, namely, *L. mesenteroides* [56.82 ± 1.5 mM, (Xu et al., 2017)] and *Bifidobacterium adolescentis* G1 [79.4 mM, (Omori et al., 2010)], for raffinose have been reported, and no *K*_m_ data of bacterial invertases for stachyose have been reported (Table 1).

### Application of InvDz13 in Saccharide Hydrolysis in Soymilk

**FIGURE 4:**
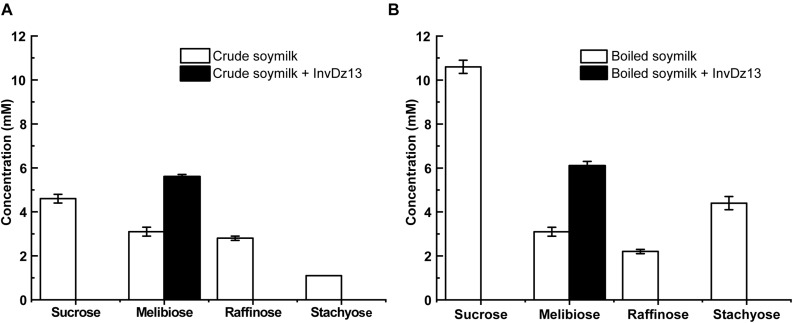
Treatment of soymilk with InvDz13. The crude soymilk **(A)** and boiled soymilk **(B)** were treated with 10 U/mL InvDz13 at 30°C for 1 h. Then the crude soymilk was further boiled for 15 min. Each soymilk sample was mixed with 70% ethanol (1:1, v/v) for 5 min, centrifuged at 20,000 × *g* to discard proteins, and determined using HPLC to detect the saccharides. Values are the means of three replication ± standard deviation.

Soymilk is a traditional food in Asian countries. It contains 8.4–30 mg/g dry matter raffinose (Garro and Savoy, 2012). The hydrolysis of raffinose-type saccharides in soymilk would reduce flatulence symptoms after drinking soymilk. Given that InvDz13 showed high activities toward sucrose and raffinose and the capability to hydrolyze stachyose, it was utilized to hydrolyze raffinose-type saccharides in soymilk. Our results showed that sucrose, melibiose, raffinose, and stachyose were present in crude and boiled soymilk (Figure 4). Boiling partially removed raffinose from soymilk. Raffinose concentration decreased from 2.8 mM to 2.2 mM after 15 min of boiling. However, boiling did not affect melibiose concentration in soymilk. By contrast, the concentrations of sucrose and stachyose increased from 4.6 to 10.6 mM and from 1.1 to 4.35 mM after 15 min of boiling, respectively, (Figure 4). InvDz13 hydrolyzed saccharides effectively in soymilk. In crude or boiled soymilk, InvDz13 hydrolyzed sucrose, raffinose, and stachyose completely within 1 h. Melibiose concentration increased from 3.1 to 5.6 mM in crude soymilk and from 3.1 to 6.1 mM in boiled soymilk (Figure 4).

Different processing techniques for the removal of raffinose from soybeans and soymilk have been investigated (Oboh et al., 2000; Medeiros et al., 2018). Among these techniques, the enzymatic hydrolysis of raffinose into sucrose and galactose by using α-galactosidases has been extensively investigated because raffinose is an α-galactosyl derivative of sucrose (Huang et al., 2018; Jang et al., 2019; Katrolia et al., 2019; Geng et al., 2020). In contrast to the strategy of using α-galactosidase, the hydrolyzation of raffinose into melibiose will increase the nutrient value of soybean products because melibiose possesses several beneficial attributes (Kaneko et al., 2004; O’Connell et al., 2013; Lee et al., 2015; Adamberg et al., 2018; Rusmini et al., 2019; Lin et al., 2020). However, no invertase has been used to treat raffinose-type saccharides in soymilk due to the lacking of suitable invertases. We confirmed that the bacterial invertase InvDz13 successfully transformed the flatulence-inducing raffinose into melibiose and doubled melibiose concentration in soymilk. Therefore, the enzymatic hydrolysis of raffinose in soymilk by using InvDz13 is practicable and may be an alternative method for improving the nutritional value of soymilk.

### Effects of Soymilk or InvDz13-Treated Soymilk on the *in vitro* Fermentation of Human Gut Microbiota

**FIGURE 5:**
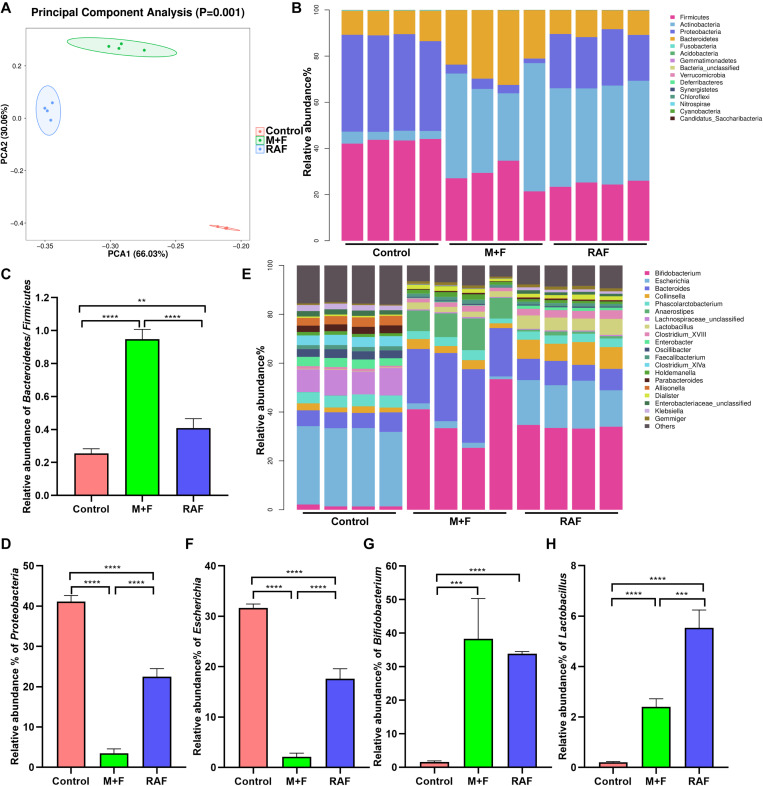
The effects of soymilk (RAF) and InvDz13-treated soymilk (M + F) on human gut microbiota via *in vitro* fermentation. **(A)** Principal component analysis. **(B)** The taxonomic composition distribution at the phylum level. **(C)** The relative abundance of phylum *Bacteroidetes* to *Firmicutes*. **(D)** The relative abundance of phylum *Proteobacteria*. **(E)** The taxonomic composition distribution at the genus level. **(F)** The relative abundance of genus *Escherichia*. **(G)** The relative abundance of genus *Bifidobacterium*. **(H)** The relative abundance of genus *Lactobacillus*. The data were analyzed using student’s *t*- test (**P < 0.01, ***P < 0.001, and ****P < 0.0001). Data show mean ± SD, *n* = 4.

*In vitro* fermentation studies on fecal consortia with soymilk (RAF group, containing raffinose) or InvDz13-treated soymilk (M + F group, wherein raffinose was hydrolyzed into melibiose and fructose) were performed to investigate the variation in human gut microbiota composition and further illustrate the nutrition-improving value of hydrolyzing raffinose-type saccharides in soymilk by using InvDz13. Interestingly, soymilk and InvDz13-treated soymilk caused significant overall structural changes in human gut microbiota (α- and β-diversity, Supplementary Figure 2 and Figure 5A). Comparison with the control revealed that both kinds of soymilk increased *Bacteroidetes* and *Actinobacteria* but reduced *Firmicutes* and *Proteobacteria* proportion (Figure 5B and Supplementary Figure 3). In detail, soymilk and InvDz13-treated soymilk increased the *Bacteroidetes*:*Firmicutes* ratio, a standard signature seen in lean and healthy phenotypes (Ridaura et al., 2013; Sharma et al., 2018), by 1.6- and 3.7-fold (Figure 5C). By contrast, the population of *Proteobacteria* in the treated samples decreased by 1.8- and 11.7-fold, respectively, compared with that in the control samples (Figure 5D).

In the control group, Proteobacteria genera, such as *Enterobacter*, *Enterobacteriaceae*, *Klebsiella*, and *Escherichia*, dominated due to their capability to metabolize amino acids as carbon and energy sources under carbohydrate-limited conditions (Figure 5E and Supplementary Figure 4; Adamberg et al., 2018). *Escherichia* abundance was reduced more significantly in the InvDz13-treated soymilk group (M + F: 2.1 ± 0.74%; *P* < 0.0001) than in the control group (RAF: 17.6 ± 1.98%, *P* < 0.0001; Figure 5F). The intake of raffinose, especially at high doses, causes flatulence in sensitive hosts due to the gas produced by gut bacteria, such as *Escherichia*, *Collinsella*, *Enterococcus*, and *Streptococcus*, during raffinose metabolism (Rey et al., 2013; Mao et al., 2018). Thus, the population of *g_Collinsella* in the samples treated with soymilk was significantly up-regulated compared with that in the control samples (*P* < 0.0001). This phenomenon was not observed in samples prepared with InvDz13-treated soymilk. Furthermore, the InvDz13-treated soymilk group had lower ratios of *g_Streptococcus* and *g_Enterococcus* than the soymilk group (*P* < 0.05; Figure 5E and Supplementary Figures 4, 5). A similar signature was observed in soymilk-treated groups: the abundances of the two reported prebiotic genera *Bifidobacterium* and *Lactobacillus* had increased dramatically (*P* < 0.0001 or *P* < 0.001) due to their capability to adhere to intestinal mucus and inhibit gastrointestinal pathogens (Figures 5G,H; Schroeder et al., 2018; Sanders et al., 2019). Furthermore, the proportion of the butyrate-producing bacteria *g_Anaerostipes*, which can stimulate prebiotic effects with *Bifidobacterium* and increase the content of acetic acid, propionic acid, and butyric acid to promote human health (Scott et al., 2014; Sanders et al., 2019), was 9.4-fold higher in the InvDz13-treated soymilk group than in the control group (*P* < 0.01; Supplementary Figure 4).

**FIGURE 6:**
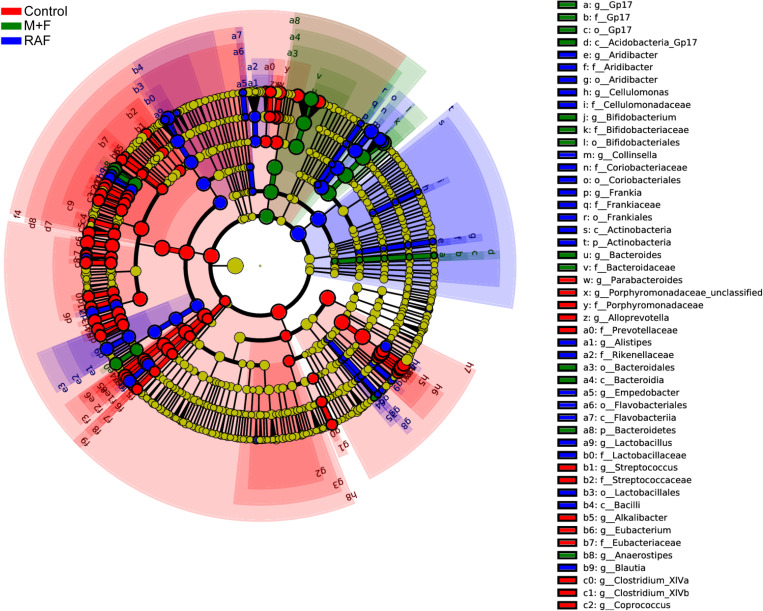
Comparisons of microbiota among Control, soymilk (RAF), and InvDz13-treated soymilk (M + F) groups based on linear discriminant analysis effect size (LEfSe). Taxa enriched in microbiota from Control (red), RAF (blue), or M + F (green) were indicated with a positive LDA score, respectively, (taxa with LDA score >2 and significance of α < 0.05 determined by Wilcoxon signed- rank test).

Specific bacteria that varied with soymilk types were detected on the basis of LEfSe. A total of 167 significantly different OTUs were identified in the three groups (Figure 6). Among these OTUs, 89, including *o_Enterobacteriales*, *p_Proteobacteria*, and *g_Escherichia*, were associated mainly with the control group. Fifty-seven OTUs, consisting of the prebiotic *g_Lactobacillus* (Zartl et al., 2018; Sanders et al., 2019) and the flatulent *g_Collinsella* (Rey et al., 2013), were highly related to the control group. However, 21 OTUs, including only prebiotic bacteria, such as *g*_*Bifidobacterium* and *g_Anaerostipes* (Scott et al., 2014; Gibson et al., 2017; Zartl et al., 2018; Sanders et al., 2019), were associated with the InvDz13-treated soymilk group. All these results proved that InvDz13 treatment can help improve the nutritional value of soymilk by increasing the proportion of beneficial bacteria, but dramatically decreased the population of gas-producing bacteria.

## Data Availability Statement

The 16s rDNA sequence information can be found in National Centre for Biotechnology Information: Submission ID: SUB8768617; BioProject SRA ID: PRJNA687351.

## Ethics Statement

Written informed consent was obtained from the individual(s) for the publication of any potentially identifiable images or data included in this article.

## Author Contributions

ZF and WP perceived the study. ZF, JL, and YX analyzed data. JL, JC, MH, WP, and CS carried out the experiments. ZF, WP, and JL wrote the manuscript. All authors commented on the manuscript.

## Conflict of Interest

KX was employed by company Anhui RenRenFu Bean Co., Ltd. The remaining authors declare that the research was conducted in the absence of any commercial or financial relationships that could be construed as a potential conflict of interest.

## Footnotes

1 https://www.ncbi.nlm.nih.gov/

2 http://www.blast.ncbi.nlm.nih.gov

3 http://smart.embl-heidelberg.de/

4 http://www.psc.edu/biomed/genedoc

5 http://www.ncbi.nlm.nih.gov/

6 http://www.cazy.org/
