# Supplementary material for: Identification of an Invertase With High Specific Activity for Raffinose Hydrolysis and Its Application in Soymilk Treatment
Source: Front Microbiol. 2021 Apr 8;12:646801. doi: 10.3389/fmicb.2021.646801 (PMC8060482; doi:10.3389/fmicb.2021.646801)
Supplement: Supplementary file 1 [file Data_Sheet_1.docx]

**Supplementary tables**

**Table S1 Bioconversion of six saccharides through InvDz13 hydrolysis.**

| **(C_6_H_10_O_5_)n** | **Substrate** | **Linkage** | **Specific Activity (U/mg)** |
| --- | --- | --- | --- |
| N=2 | Sucrose | O-*α*-D-glucopyranosyl-(1-2)-O-*β*-D-fructofuranoside | 225 |
|  | Cellobiose | O-*β*-D-glucopyranosyl-(1-4)-O-D-glucopyranoside | 0 |
|  | Maltose | O-*α*-D-glucopyranosyl-(1-4)-O-D-glucopyranoside | 0 |
|  | Lactose | O-*β*-D-galactopyranosyl-(1-4)-D-glucopyranoside | 0 |
| N=3 | Raffinose | O-*α*-D-galactopyranosyl-(1-6)-O-*α*-D-glucopyranosyl-(1-2)-O-*β*-D-fructofuranoside | 229 |
| N=4 | Stachyose | O-*α*-D-galactopyranosyl-(1-6)-O-*α*-D-galactopyranosyl-(1-6)-O-*α*-D-glucopyranosyl-(1-2)-O-*β*-D-fructofuranoside | 24 |

The enzymatic reactions were performed at pH 6.5 and 35 ^o^C.

**Supplementary figures**

**Figure S1**


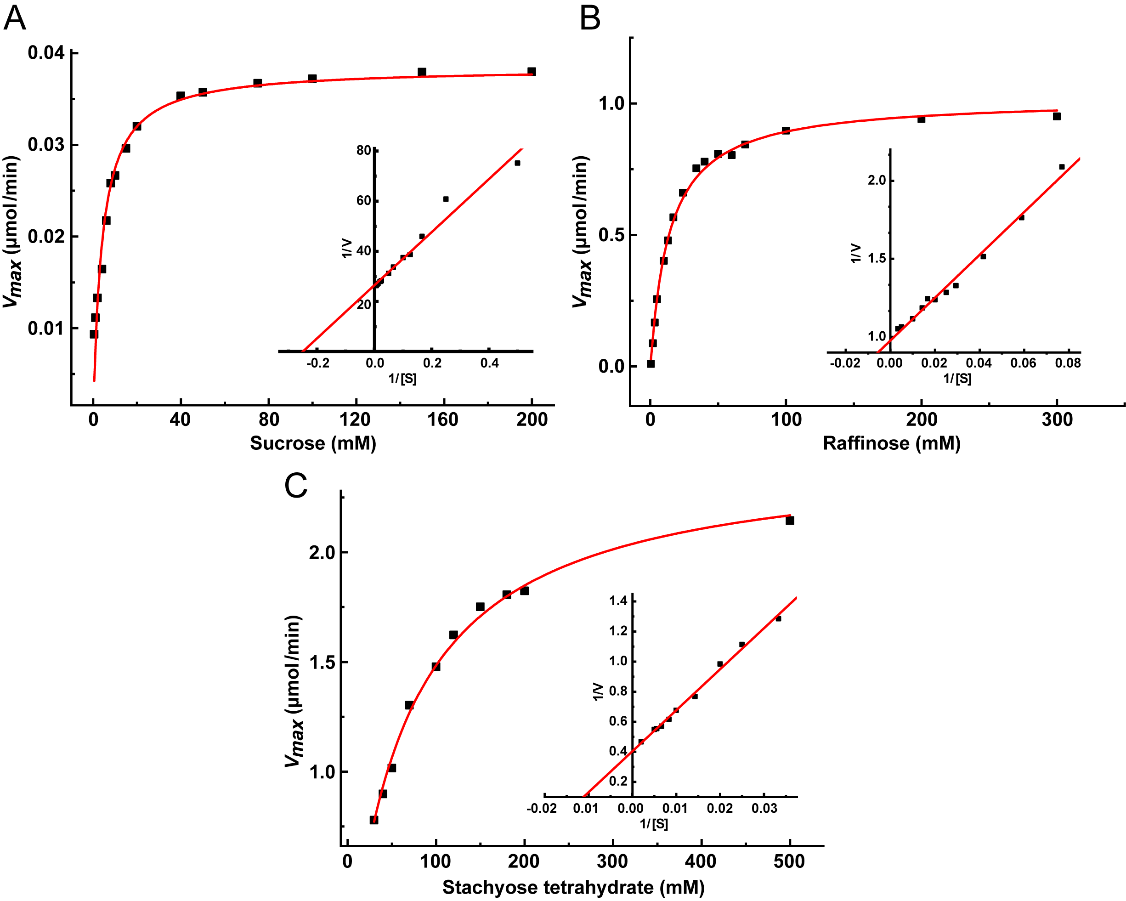


**Figure S1 The kinetic constants of InvDz13 on sucrose (A), raffinose (B), and stachyose (C).** The reaction was carried out by incubating the enzyme in 50 mM citrate-phosphate buffer (pH 6.5) containing sucrose, raffinose, or stachyose at a concentration range of 1 mM-1,000 mM at 35 °C for 5 min.

**Figure S2**

**
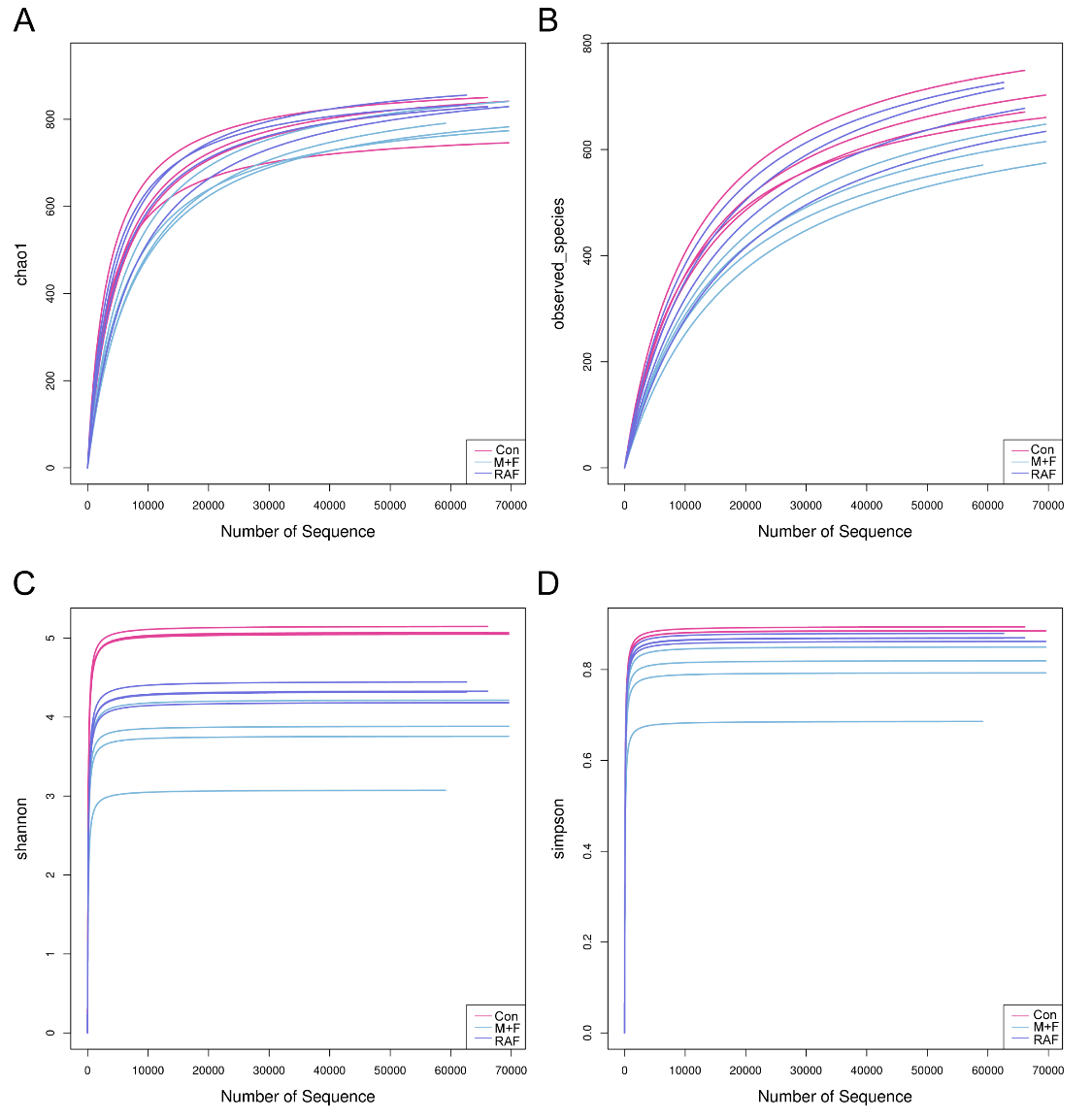
**

**Figure S2 The *α*- diversity of the human gut microbiota in soymilk (RAF) and InvDz13-treatedsoymilk (M+F) via fermentation *in vitro*.** (A) Chao 1 index. (B) observed OTUs. (C) Shanon index. (D) Simpson index.

**Figure S3**


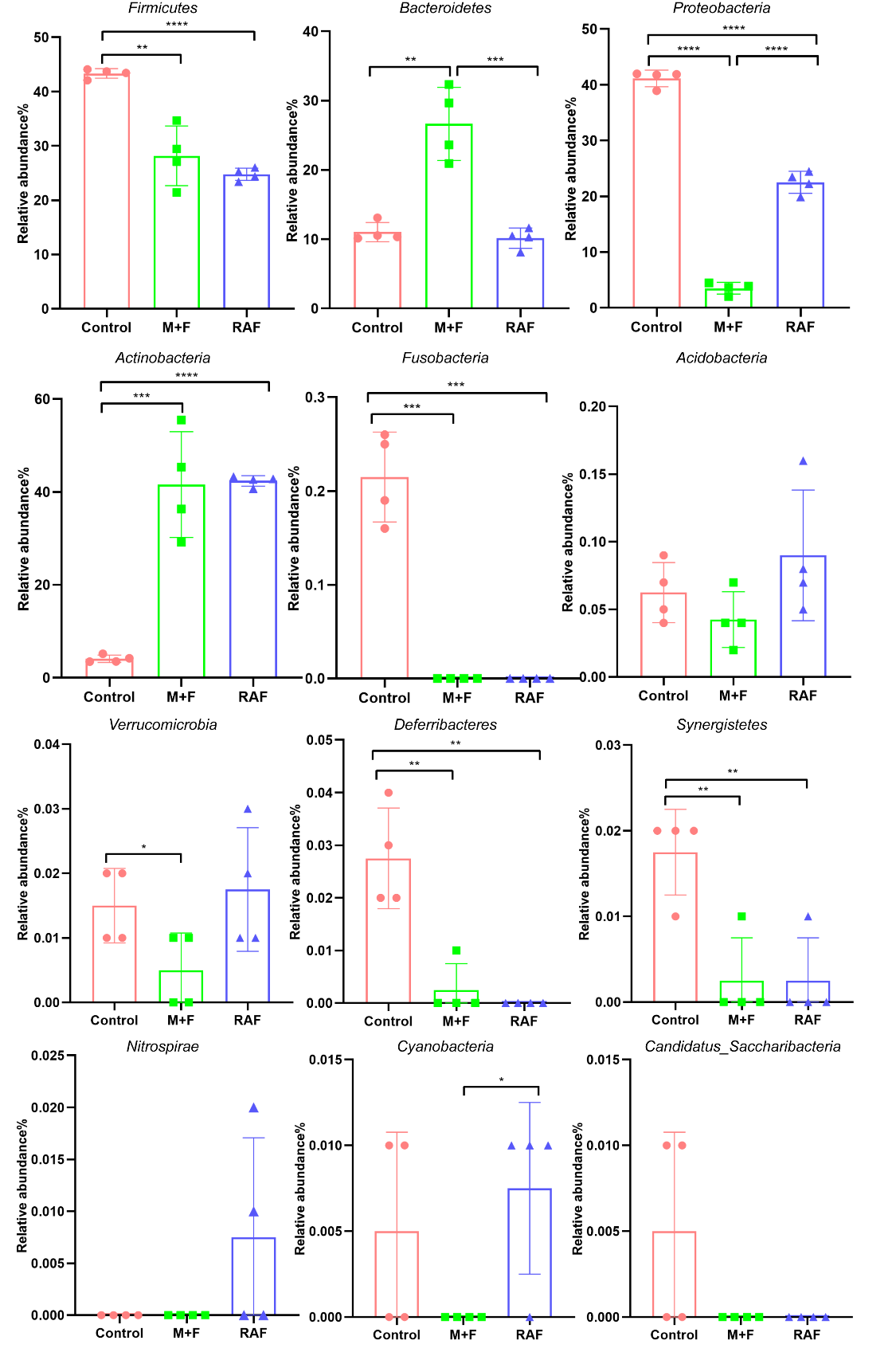


**Figure S3 Effects of soymilk (RAF) and InvDz13-treated soymilk (M+F) on the human gut microbiota at the phylum level *in vitro*.** Values were expressed as means ± SD (n = 4). * *P* < 0.05, ** *P* < 0.01, *** *P* < 0.001, **** *P* < 0.0001.

**Figure S4**


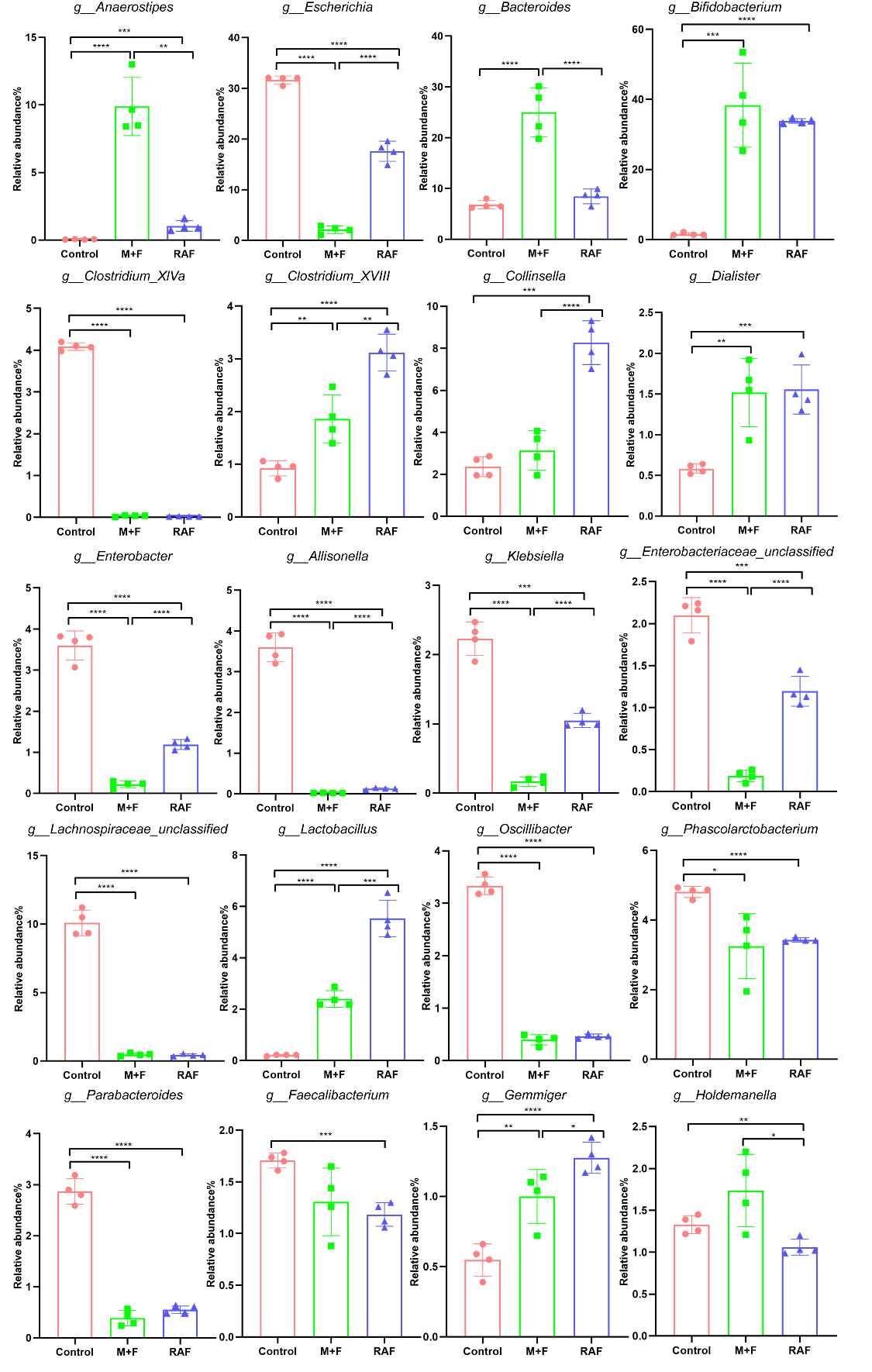


**Figure S4** **Effects of soymilk (RAF) and InvDz13-treated soymilk (M+F) on the top 20 gut bacteria at the genus level *in vitro*.** Values were expressed as means ± SD (n = 4). * *P* < 0.05, ** *P* < 0.01, *** *P* < 0.001 and **** *P* < 0.0001.

**Figure S5**


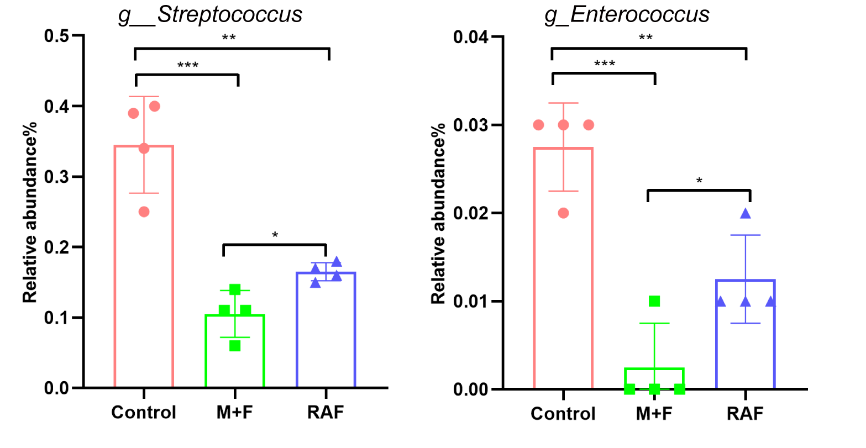


**Figure S5** **Effects of soymilk (RAF) and InvDz13-treated soymilk (M+F) on two gas produced bacteria *g*_*Enterococcus* and *g_Streptococcus* *in vitro*.** Values were expressed as means ± SD (n = 4). * *P* < 0.05, ** *P* < 0.01 and *** *P* < 0.001.
